# Long-term non-pharmacological weight loss interventions for adults with type 2 diabetes mellitus

**DOI:** 10.1590/1516-3180.20161342T2

**Published:** 2015-03-17

**Authors:** Susan L. Norris, Xuanping Zhang, Alison Avenell, Edward Gregg, Tamara Brown, Christopher H. Schmid, Joseph Lau

## Abstract

**BACKGROUND:**

Most persons with type 2 diabetes are overweight and obesity worsens the metabolic and physiologic abnormalities associated with diabetes.

**OBJECTIVE:**

The objective of this review is to assess the effectiveness of lifestyle and behavioral weight loss and weight control interventions for adults with type 2 diabetes.

**METHODS:**

*Search methods:* Studies were obtained from computerized searches of multiple electronic bibliographic databases, supplemented with hand searches of selected journals and consultation with experts in obesity research.

*Selection criteria:* Studies were included if they were published or unpublished randomized controlled trials in any language, and examined weight loss or weight control strategies using one or more dietary, physical activity, or behavioral interventions, with a follow-up interval of at least 12 months.

*Data collection and analysis:* Effects were combined using a random effects model.

**MAIN RESULTS:**

The 22 studies of weight loss interventions identified had a 4,659 participants and follow-up of 1 to 5 years. The pooled weight loss for any intervention in comparison to usual care among 585 subjects was 1.7 kg (95 % confidence interval [CI] 0.3 to 3.2), or 3.1% of baseline body weight among 517 subjects. Other main comparisons demonstrated non significant results: among 126 persons receiving a physical activity and behavioral intervention, those who also received a very low calorie diet lost 3.0 kg (95% CI -0.5 to 6.4), or 1.6% of baseline body weight, more than persons receiving a low-calorie diet. Among 53 persons receiving identical dietary and behavioral interventions, those receiving more intense physical activity interventions lost 3.9 kg (95% CI -1.9 to 9.7), or 3.6% of baseline body weight, more than those receiving a less intense or no physical activity intervention. Comparison groups often achieved significant weight loss (up to 10.0 kg), minimizing between-group differences. Changes in glycated hemoglobin generally corresponded to changes in weight and were not significant when between-group differences were examined. No data were identified on quality of life and mortality.

**AUTHORS CONCLUSIONS:**

Weight loss strategies using dietary, physical activity, or behavioral interventions produced small between-group improvements in weight. These results were minimized by weight loss in the comparison group, however, and examination of individual study arms revealed that multicomponent interventions including very low calorie diets or low calorie diets may hold promise for achieving weight loss in adults with type 2 diabetes.

The full text is available free-of-charge from: http://onlinelibrary.wiley.com/doi/10.1002/14651858.CD004095.pub2/abstract
